# Prevalence of psychological comorbidities and their association with injection therapy outcomes in patients with chronic low back pain: a registry-based retrospective cohort study

**DOI:** 10.3389/fmed.2026.1752707

**Published:** 2026-04-28

**Authors:** Anas Afifi, Khaled Allan, Marc Brügmann, Andrea Roth-Daniek, Rolf Sobottke, Moh’d Yazan Khasawneh, Koroush Kabir, Alba Shehu, Michel Teuben, Ümit Mert, Maher Ghandour, Mohamad Agha Mahmoud

**Affiliations:** 1 Department of Orthopedics and Trauma Surgery, Katholisches Klinikum Bochum - St. Josef Hospital, Ruhr University Bochum, Bochum, Germany; 2 Department of Spine, Neuro- and Orthopedic Surgery, Rhein-Maas Clinic, Würselen, Germany; 3 Department of Pain Therapy and Palliative Care, Medical Centre Aachen GmbH, Würselen, Germany; 4 Department of Trauma and Orthopedic Surgery, Helios University Hospital Wuppertal, University of Witten/Herdecke, Wuppertal, Germany; 5 Department of Traumatology, University Hospital Zürich, Zürich, Switzerland

**Keywords:** anxiety, chronic low back pain, chronic pain disorder, depression, injection therapy, radiculopathy

## Abstract

**Background:**

Chronic low back pain (CLBP), with or without radicular pain (RP), is a major cause of disability and reduced quality of life. Psychological comorbidities such as depression, anxiety, and chronic pain disorder are common in this population and may influence pain perception and treatment outcomes. This study aimed to assess the prevalence of psychological disorders in patients with CLBP and RP and to evaluate their association with pain severity and response to lumbar injection therapy.

**Methods:**

This retrospective cohort study included 963 patients with chronic lumbar pain refractory to conservative treatment and without surgical indications. Psychological conditions were assessed using validated instruments incorporated into the German Pain Questionnaire (DSF), including DIPS, HADS-D, and DASS-21. Pain severity was measured using the Numerical Rating Scale (NRS) before and after injection therapy. Associations between psychological comorbidities and treatment outcomes were analyzed.

**Results:**

Depression was identified in 37.6% of patients using DIPS, with prevalence estimates of 41 and 31% using HADS and DASS, respectively. Anxiety was present in 28–31% of patients, and stress symptoms in 45%. Chronic pain disorder with somatic and psychological factors (F45.41) was diagnosed in 17.1%. Patients with psychological comorbidities reported higher baseline NRS scores and experienced smaller reductions in pain following treatment. For example, patients with depression had higher baseline back pain scores than those without depression (8.15 vs. 7.24) and higher post-treatment scores (4.37 vs. 3.05, *p* < 0.01). Similar patterns were observed for anxiety and chronic pain disorder.

**Conclusion:**

Psychological comorbidities are highly prevalent in patients with CLBP and RP and are associated with greater pain severity and reduced improvement following injection therapy. These findings support the integration of psychological assessment and intervention into multidisciplinary management strategies for CLBP.

## 1 Introduction

Chronic pain is the leading cause of disability-adjusted life years (DALYs) among all chronic medical conditions and the primary contributor to work-related disability, resulting in substantial economic burdens. Among these conditions, chronic low back pain (CLBP), with or without radicular pain (RP) due to degenerative changes in the lumbar spine, is a major source of morbidity and functional limitations (1–3).

The management of CLBP necessitates a multifaceted approach that integrates diverse therapeutic modalities aimed at optimizing patient outcomes (4–6). A growing body of evidence, including systematic reviews, supports the effectiveness of multidisciplinary treatment programs in reducing pain intensity and improving functional capacity in patients with CLBP (7–9). Notably, the incorporation of psychological assessment and interventions within treatment protocols has shown promise in enhancing clinical outcomes (10).

Psychological and emotional factors play a significant role in the experience and progression of CLBP and RP, often exacerbating pain perception and contributing to functional impairment (11–13). Mental health conditions such as depression and anxiety are associated with greater pain intensity and poorer physical functioning (14). Preoperative depression has been linked to worse outcomes following lumbar spine surgery (15–17). However, there remains a paucity of research examining the impact of psychological comorbidities (e.g., depression and anxiety) on outcomes after lumbar infiltration therapies, especially with respect to long-term follow-up (10, 12, 18, 19).

This study aimed to determine the prevalence of psychological disorders—specifically depression, anxiety, and chronic pain disorder—and to evaluate their influence on patient-reported pain severity, measured by the numerical rating scale (NRS), following lumbar infiltration therapies. These findings are intended to inform clinical decision-making and improve individualized treatment strategies for patients with CLBP and RP. It should be noted that the psychological assessment performed in this study served as a characterization tool and did not inform the decision to administer injection therapy; the presence or absence of a psychological disorder was neither an inclusion nor an exclusion criterion.

## 2 Materials and methods

### 2.1 Study design

This retrospective cohort study was conducted at the Conservative Spine/Back Centre, under the supervision of the Department of Spine Surgery (accredited by the German Spine Society (DWG) and the EUROSPINE Society) and Pain Management and Palliative Medicine. Data were collected prospectively from our institutional database. Owing to the observational retrospective design, the study evaluates associations between psychological comorbidities and treatment outcomes without establishing causal relationships. Prior to elective admission for injection therapy, all patients underwent clinical evaluation by a spine surgeon and lumbar spine MRI to rule out surgical indications. During the inpatient stay, patients received concurrent psychological counseling provided by a qualified psychologist as an integral component of the multidisciplinary treatment program. Ethical approval for the scientific work based on the German Spine Society (DWG) Registry at Rhein-Maas Hospital was granted by the Ethics Committee of the Medical Association of North Rhine (Ärztekammer Nordrhein), reference number 2016448. Informed consent was obtained from all participants prior to any form of injection therapy.

### 2.2 Study population

Adults were eligible for inclusion if they met all of the following criteria:

- Chronic lumbar pain and/or radicular leg pain persisting despite at least 6 months of maximal conservative therapy (including oral analgesics, physiotherapy and lifestyle modification, conservative treatment prior to referral consisted primarily of oral analgesic medication and physiotherapy),
- Degenerative changes on lumbar spine imaging consistent with clinical findings,
- Absence of a definitive indication for spinal surgery.

Exclusion criteria included:

- Confirmed surgical indication,
- Spinal tumors,
- Vertebral fractures,
- Lack of written informed consent.

A history of psychiatric or psychological disorders was not an exclusion criterion. Patients with known psychological comorbidities were included and their conditions were characterized using the DSF as part of routine clinical assessment. The presence of psychological disorders was neither a criterion for, nor a contraindication to, injection therapy.

### 2.3 Injection therapy

Injection therapy was performed as part of routine clinical management at the Conservative Spine/Back Centre following multidisciplinary evaluation by a spine surgeon and pain specialist. Patients were selected for interventional treatment after failure of conservative therapy and in the absence of surgical indications. The specific type and technical details of injection procedures were determined individually based on clinical presentation and imaging findings according to institutional practice. As this was a registry-based analysis, detailed procedural parameters such as the exact injection technique, number of procedures, and treated spinal levels were not consistently available for all patients.

### 2.4 Outcome parameters

Patient-reported outcomes were collected using the paper-based German Pain Questionnaire (Deutsche Schmerzfragebogen-DSF). All patients underwent at least one psychological assessment session lasting a minimum of 50 min, conducted jointly by the patient and a qualified psychologist as part of the concurrent psychological counseling program. In cases where the psychologist deemed further clarification or more thorough evaluation necessary—based on the patient’s psychological complexity or level of engagement—additional sessions were conducted at the psychologist’s discretion.

The DSF, developed by the German pain Society, is a validated instrument used for evaluating chronic pain, including its intensity, duration, psychological comorbidities, and functional impact. It also assists clinicians in developing individualized treatment plans (20, 21).

The following variables were analyzed:

- Patient demographics (gender and age).
- Psychological comorbidities (depression, anxiety, stress, chronic pain disorder with somatic and psychological factors (ICD-10: F45.41))
- Pain severity [Numerical Rating Scale (NRS)].
- Influence of injection modalities on NRS pain severity across psychological subgroups.

Pain severity using the Numerical Rating Scale (NRS) was recorded before injection therapy and at the time of post-treatment clinical assessment during the same treatment episode, as documented in the registry database. Length of hospital stay was recorded as a descriptive measure characterizing the inpatient treatment episode. Given the elective inpatient nature of the treatment program, stay duration reflects logistical and administrative aspects of care delivery rather than constituting a primary or secondary outcome measure.

### 2.5 Psychological assessment tools

#### 2.5.1 DIPS-depression: (diagnostisches interview bei psychischen Störungen)

Part of the structured DIPS interview system, this tool assesses depressive symptoms on a defined scale (typically 0–3 or 0–4), with higher scores indicating greater severity (22).

#### 2.5.2 HADS-D: (hospital anxiety and depression scale—German version)

HADS is widely used to screen for anxiety and depression in patients with physical health conditions. The German version (HADS-D) maintains cultural and linguistic validity. Each subscale (anxiety and depression) includes 7 items scored from 0–21; a score of ≥8 suggests clinically relevant symptoms. The full scale (14 items) yields a combined score ranging from 0 to 42 (23–25). Notably, as of 2015, HADS was replaced in the DFS by the DASS-21 (Depression, Anxiety, and Stress Scale, 21 items).

#### 2.5.3 DASS-21: (depression, anxiety, and stress scale—21 items)

DASS-21 is a validated instrument for measuring depression, anxiety, and stress. It includes 21 items, with 7 items per subscale, each scored on a 4-point Likert scale (0–3). Higher scores reflect greater psychological distress (26, 27). Thresholds for clinical relevance were:

- Depression (D): ≥10
- Anxiety (A): ≥6
- Stress (S): ≥10

### 2.6 Statistical analysis

Statistical analysis was performed using SPSS for Windows 22.0 (IBM Corp., Chicago, IL, United States). The differences between groups were calculated with Chi-square or Fisher’s exact test for the ordinal data and T-tests or the Mann–Whitney U test for the continuous data. *p*-values< 0.05 were considered to be statistically significant.

## 3 Results

### 3.1 Baseline characteristics

**Figure 1:**
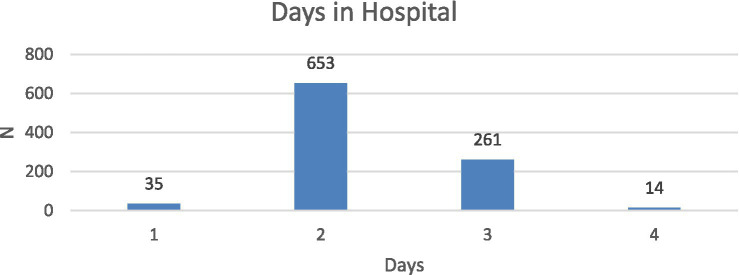
Distribution of length of hospital stay (days).

Between January 1, 2015 and December 31, 2018, a total of 963 patients were treated as elective inpatients at the Conservative Spine/Back Centre. Patient ages ranged from 23 to 90 years (mean = 63.53 years, SD = 13.02), with no significant age-related differences in psychological variables. The cohort included 587 females and 376 males, with mean ages of 65.72 and 61.45 years, respectively. Gender was not associated with significant differences in psychological outcomes. Length of hospitalization ranged from 1 to 8 days (mean = 3.27; SD = 0.63), and its distribution is shown in Figure 1.

### 3.2 Prevalence of depression

**Figure 2:**
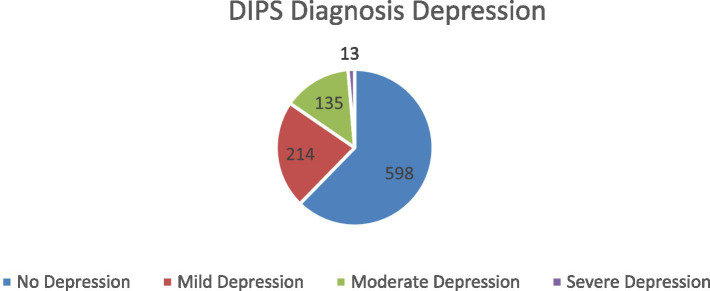
Distribution of depression scores based on the DIPS diagnostic interview.

Depressive symptoms assessed using the DIPS diagnostic interview were present in 37.6% of patients (Figure 2). Prevalence estimates differed across assessment tools, with rates of 31% using the DASS-21 depression scale and 41% using HADS-D.

**Figure 3:**
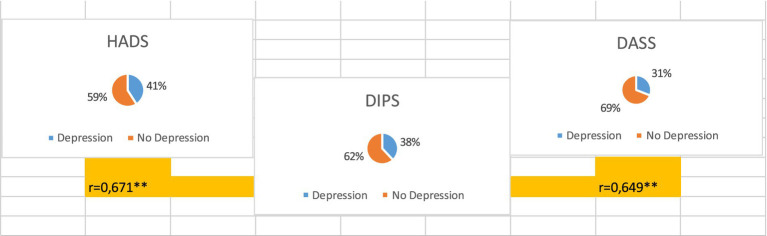
Comparison of depression assessment tools (DIPS, DASS-21, and HADS) and correlations between scores.

**Figure 4:**
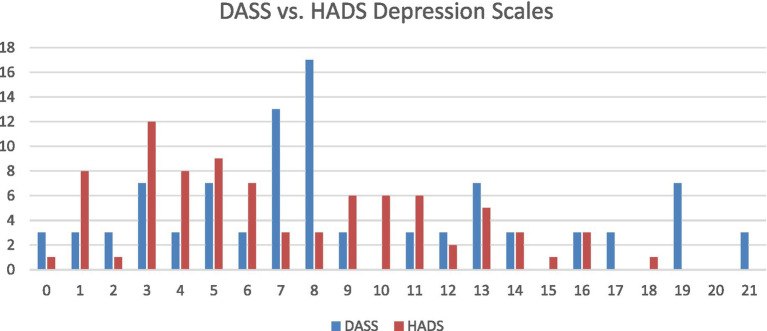
Correlation between DASS-21 and HADS depression scores.

Depression scores showed strong correlations between DIPS and DASS (*r* = 0.649) and between DIPS and HADS-D (*r* = 0.671) (Figure 3). HADS-D scores ranged from 0 to 18 (mean = 7.3; SD = 4.5), and DASS depression scores ranged from 0 to 21 (mean = 9.0; SD = 5.6). The relationship between DASS and HADS-D scores is presented in Figure 4.

### 3.3 Prevalence of anxiety

**Figure 5:**
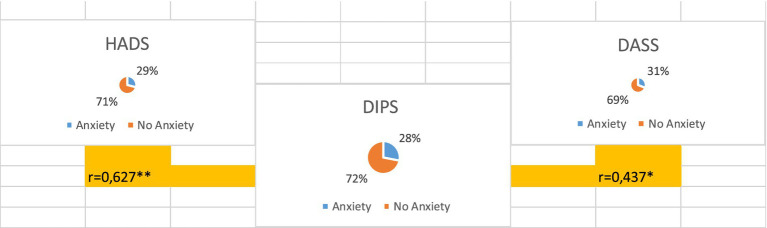
Prevalence of anxiety in the study cohort across assessment tools.

Anxiety prevalence was 29% using HADS-A, 31% using DASS-21, and 28% using DIPS. Moderate correlations were observed between HADS-A and DIPS (*r* = 0.627) and between DASS anxiety and DIPS (*r* = 0.437). Results across assessment tools are summarized in Figure 5.

### 3.4 Prevalence of stress

**Figure 6:**
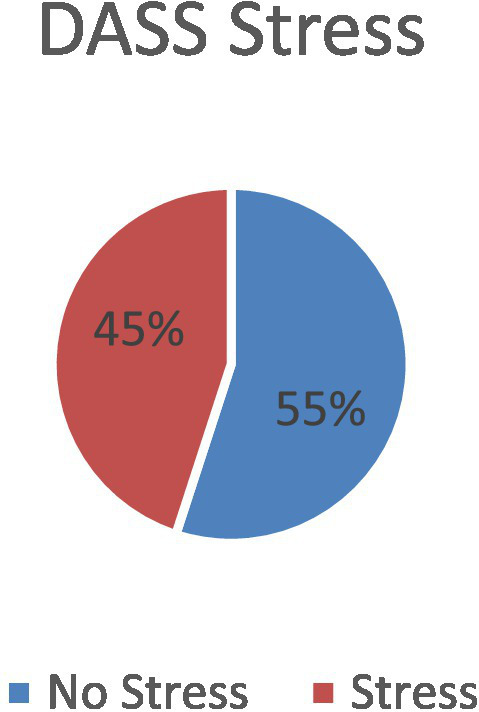
Prevalence of stress based on DASS-21 assessment.

Based on DASS-21 assessment, 45% of patients exhibited stress symptoms (Figure 6).

### 3.5 Prevalence of chronic pain disorder (F45.41)

**Figure 7:**
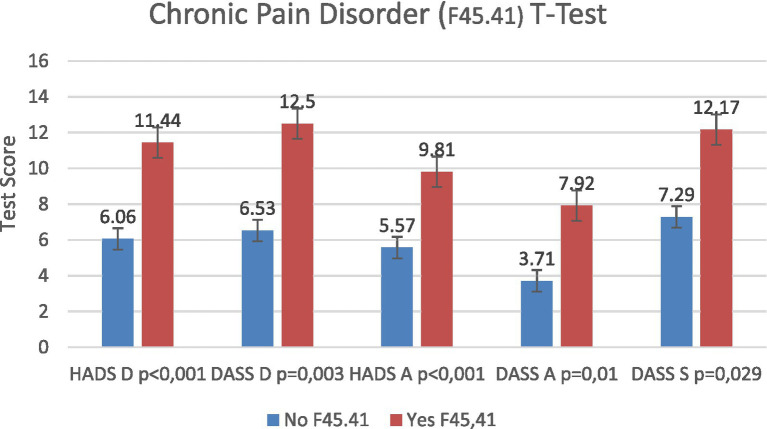
Pain and psychological assessment outcomes in patients with chronic pain disorder (ICD-10: F45.41).

Chronic pain disorder with somatic and psychological factors (ICD-10: F45.41) was diagnosed in 164 patients (17.1%). This diagnosis was significantly associated with multiple DSF subscale scores (Table 1). Patients with chronic pain disorder demonstrated significantly higher scores across DSF subscales, indicating greater perceived pain severity (Figure 7).

**Table 1:** The correlation between chronic pain disorder and DSF subscales.

		Correlation				
		HADS depression	DASS depression	HADS anxiety	DASS anxiety	DASS stress
Chronic pain Disorder F45.41	Correlations according to Pearson	0.507^**^	0.534^**^	0.400^**^	0.472^**^	0.405^*^
	Significance	0	0.003	0.001	0.01	0.029
	(2-tailed)					

*0.05 significance (2-tailed); **0.01 significance (2-tailed).

### 3.6 Therapeutic outcomes: pain severity (NRS)

#### 3.6.1 Overall effect of injection therapy

**Figure 8:**
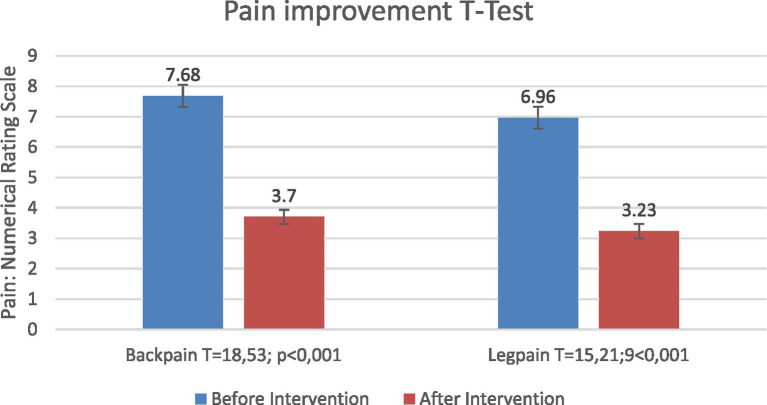
Changes in back and leg pain severity (NRS) before and after injection therapy in the overall cohort.

Injection therapy was associated with significant reductions in both back and leg pain severity. Mean back pain NRS decreased from 7.69 at baseline to 3.70 post-treatment, and leg pain NRS decreased from 6.96 to 3.23 (Back: *T* = 18.53; *p* < 0.001; Leg: *T* = 15.21; *p* < 0.001). Overall changes are shown in Figure 8.

#### 3.6.2 Pain severity by depression status

**Figure 9:**
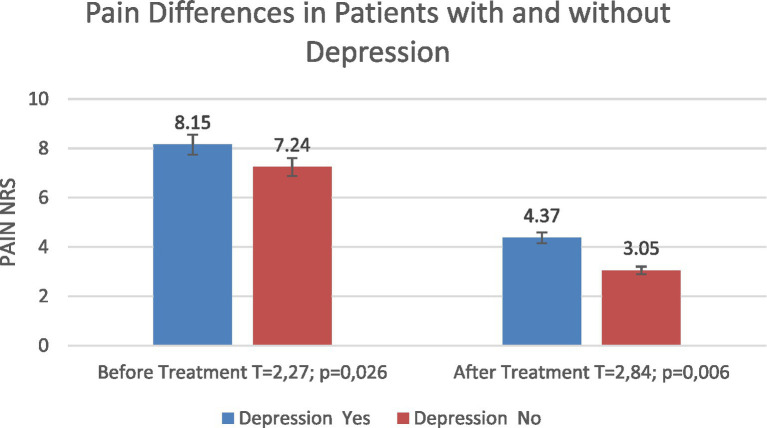
Pain severity (NRS) in patients with and without depression before and after treatment.

Patients with depression reported higher baseline back pain compared with those without depression (8.15 vs. 7.24). Following treatment, NRS scores decreased to 4.37 in patients with depression and 3.05 in those without. Differences were significant both before and after treatment (*T* = 2.27; *p* = 0.026 and *T* = 2.84; *p* = 0.006, respectively) (Figure 9).

#### 3.6.3 Pain severity by anxiety status

**Figure 10:**
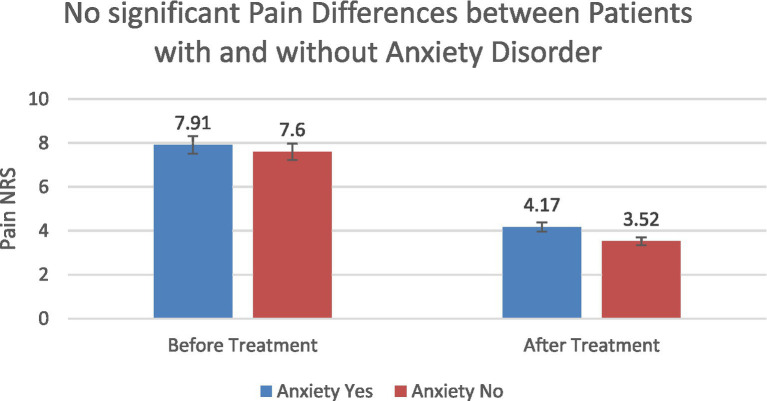
Pain severity (NRS) according to anxiety status before and after treatment.

Patients with anxiety showed slightly higher baseline NRS scores than those without anxiety (7.91 vs. 7.60). Post-treatment scores decreased to 4.17 and 3.52, respectively, with no statistically significant differences between groups (Figure 10).

#### 3.6.4 Pain severity by chronic pain disorder status (F45.41)

**Figure 11:**
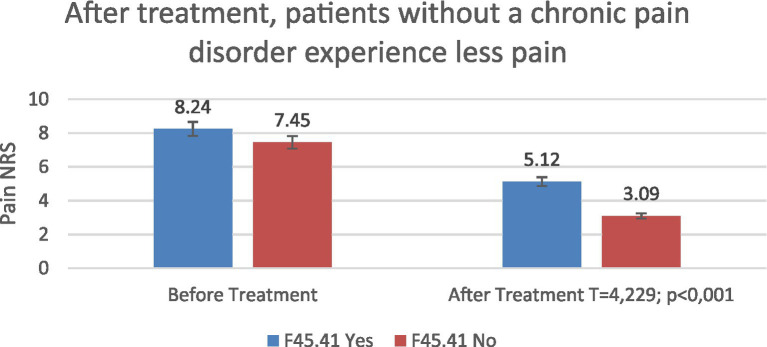
Pain severity (NRS) according to chronic pain disorder status (ICD-10: F45.41) before and after treatment.

Baseline back pain NRS was higher in patients with chronic pain disorder compared with those without (8.24 vs. 7.45). After treatment, scores decreased to 5.12 and 3.09, respectively, with significantly greater pain reduction observed in patients without chronic pain disorder (*T* = 4.23; *p* < 0.001) (Figure 11).

## 4 Discussion

This study evaluated the prevalence and clinical impact of psychological comorbidities in patients with CLBP with or without radicular pain (RP) undergoing injection therapy at a tertiary Conservative Spine/Back Centre. All patients had failed conservative management and had no indication for surgery. Consistent with existing evidence, psychological disorders—including depression, anxiety, and stress—were highly prevalent and were associated with increased pain severity and reduced treatment response.

The German Pain Questionnaire (DSF) served as the primary assessment tool, integrating validated instruments including DIPS, HADS-D, and DASS-21. The use of multiple standardized measures enabled comprehensive evaluation of psychological symptoms and demonstrated strong concordance between assessment tools, particularly for depressive symptoms (28–31).

A high prevalence of psychological disturbances was observed in this cohort. Depression was identified in 37.6% of patients using DIPS, with comparable prevalence estimates using HADS (41%) and DASS (31%). These rates substantially exceed the reported prevalence in the general German population (8.1%) (32), highlighting the considerable psychological burden among patients with CLBP. Similarly, anxiety was present in 28–31% of patients, approximately twice the prevalence reported in the general population (15.3%) (33), and stress symptoms were detected in 45% of the cohort. Chronic pain disorder with somatic and psychological factors (ICD-10: F45.41) was diagnosed in 17.1% of patients and showed strong associations with DSF subscale scores (34, 35).

Psychological comorbidities were consistently associated with higher baseline pain intensity. Patients with depression, anxiety, and chronic pain disorder reported greater baseline back pain compared with those without these conditions. Although injection therapy significantly reduced pain across the cohort, patients with psychological comorbidities experienced smaller improvements, particularly those with depression and chronic pain disorder. These findings suggest that psychological comorbidities are associated with reduced treatment response in patients undergoing interventional pain management. Whether this reflects a causal relationship, shared underlying mechanisms, or unmeasured confounders cannot be determined from the present observational design.

The observed associations may reflect mechanisms such as altered pain perception, central sensitization, maladaptive coping strategies, and interactions between psychological and somatic symptoms (10, 12, 15–19). These findings support the biopsychosocial model of chronic pain and highlight the importance of addressing psychological factors in treatment planning.

These findings are consistent with a growing body of evidence supporting the biopsychosocial model of chronic pain (7, 8). Multidisciplinary treatment programs that incorporate psychological interventions have demonstrated superiority over unimodal approaches in reducing pain intensity and improving functional capacity in patients with CLBP (7, 8). Our findings extend this evidence base to the interventional pain setting, showing that even after injection therapy, psychological comorbidities are associated with clinically meaningful differences in pain outcomes. This is broadly consistent with evidence that preoperative depression is associated with inferior outcomes after lumbar spine surgery (15–17), and suggests that similar mechanisms—including altered pain perception, central sensitization, and maladaptive coping—operate across the spectrum of interventional and surgical pain management (10, 12). Few studies have specifically examined the influence of psychological comorbidities on outcomes following lumbar injection therapies, and psychosocial variables remain underreported in the interventional pain literature (36, 37). Emerging evidence suggests that psychological distress and other psychosocial factors may influence treatment response and post-injection pain outcomes (38, 39). While preoperative depression has been consistently associated with poorer outcomes after lumbar spine surgery (18), its role in conservative interventional treatments remains less well characterized. The present findings contribute to growing evidence supporting the integration of psychological assessment and support within multidisciplinary pain management strategies to optimize treatment outcomes and guide patient selection (12).

Several limitations should be acknowledged. First, the retrospective observational design precludes causal inference; all findings should be interpreted as associations. Critically, the pre-morbid psychological status of patients was unavailable; consequently, the temporal sequence of psychological disorders and chronic pain cannot be established. Second, detailed procedural parameters of injection therapy—including specific techniques, number of procedures, and treated spinal levels—were not consistently available within the registry dataset, limiting reproducibility and evaluation of treatment heterogeneity. Third, post-treatment pain assessment was based on clinical follow-up data from the same inpatient episode; standardized medium- and long-term outcomes were not assessed, restricting conclusions regarding the durability of treatment effects. Fourth, concurrent psychological counseling was provided during the inpatient stay, and its independent contribution to the observed pain outcomes cannot be disentangled from the effect of injection therapy alone. Fifth, several clinically important confounders—including pain duration, functional disability, opioid and analgesic use, socioeconomic and educational status, sleep disturbance, and pain catastrophizing—were not systematically recorded and could not be adjusted for in the analysis. Sixth, rheumatological (e.g., ankylosing spondylitis) and neurological (e.g., Parkinson’s disease) comorbidities, which are independently associated with elevated rates of psychological disorders, were not used as exclusion criteria and may have contributed to the observed psychological burden. Future prospective studies with standardized interventional protocols, comprehensive psychosocial assessment, and long-term follow-up are strongly warranted.

Overall, this study demonstrates a high prevalence of psychological comorbidities among patients with CLBP and RP and their association with pain severity and treatment response. These findings support a multidisciplinary approach that integrates psychological and physical components of care to improve outcomes in chronic pain management.

## 5 Conclusion

Psychological comorbidities—including depression, anxiety, and chronic pain disorder—are highly prevalent in patients with chronic low back pain and are associated with greater baseline pain severity and reduced improvement following injection therapy. Although interventional treatment effectively reduced pain overall, psychological factors were associated with diminished treatment response. These findings support the incorporation of psychological assessment and intervention into multidisciplinary management strategies for patients with CLBP to optimize clinical outcomes.

## Data Availability

The raw data supporting the conclusions of this article will be made available by the authors, without undue reservation.

## References

[1] Cieza A Causey K Kamenov K Hanson SW Chatterji S Vos T. Global estimates of the need for rehabilitation based on the global burden of disease study 2019: a systematic analysis for the global burden of disease study 2019. Lancet. (2021) 396:2006–17. doi: 10.1016/s0140-6736(20)32340-0, 33275908 PMC7811204

[2] Waddell G Burton AK. Occupational health guidelines for the Management of low Back Pain at work: evidence review. Occupat Med. (2001) 51:124–35. doi: 10.1093/occmed/51.2.124, 11307688

[3] James SL Abate D Abate KH Abay SM Abbafati C Abbasi N . Global, regional, and national incidence, prevalence, and years lived with disability for 354 diseases and injuries for 195 countries and territories, 1990-2017: a systematic analysis for the global burden of disease study 2017. Lancet. (2018) 392:1789–858. doi: 10.1016/s0140-6736(18)32279-7 30496104 PMC6227754

[4] Filippiadis DK Kelekis A. A review of percutaneous techniques for low Back pain and neuralgia: current trends in epidural infiltrations, intervertebral disk and facet joint therapies. Br J Radiol. (2016) 89:20150357. doi: 10.1259/bjr.20150357, 26463233 PMC4985947

[5] Bailly F Trouvin AP Bercier S Dadoun S Deneuville JP Faguer R . Clinical guidelines and care pathway for management of low back pain with or without radicular pain. Joint Bone Spine. (2021) 88:105227. doi: 10.1016/j.jbspin.2021.105227, 34051387

[6] Ho EK-Y Chen L Simic M Ashton-James CE Comachio J Wang DXM . Psychological interventions for chronic, non-specific low back pain: systematic review with network meta-analysis. BMJ. (2022) 376:e067718. doi: 10.1136/bmj-2021-067718 35354560 PMC8965745

[7] Kamper SJ Apeldoorn AT Chiarotto A Smeets RJ Ostelo RW Guzman J . Multidisciplinary biopsychosocial rehabilitation for chronic low Back pain. Cochrane Database Syst Rev. (2014) 2014:CD000963. doi: 10.1002/14651858.CD000963.pub3, 25180773 PMC10945502

[8] Patrick LE Altmaier EM Found EM. Long-term outcomes in multidisciplinary treatment of chronic low back pain: results of a 13-year follow-up. Spine. (2004) 29:850–5. doi: 10.1097/00007632-200404150-00006, 15082983

[9] Koes BW van Tulder M Lin CW Macedo LG McAuley J Maher C. An updated overview of clinical guidelines for the Management of non-Specific low Back Pain in primary care. Eur Spine J. (2010) 19:2075–94. doi: 10.1007/s00586-010-1502-y, 20602122 PMC2997201

[10] Zackova M Aspide R Braghittoni A Zenesini C Palandri GJESJ. Yellow flag on prognostic factors for non-specific chronic low back pain patients subjected to mini-invasive treatment: a cohort study. Eur Spine J. (2020) 29:1879–86. doi: 10.1007/s00586-020-06475-8, 32495278

[11] Marčić M Mihalj M Ivica N Pintarić I Titlić M. How severe is depression in low back pain patients? Acta Clin Croat. (2014) 53:267–71. 25509235

[12] Oliveira DS Vélia Ferreira Mendonça L Sofia Monteiro Sampaio R Manuel Pereira Dias Castro-Lopes J Ribeiro Azevedo LF The impact of anxiety and depression on the outcomes of chronic low back pain multidisciplinary pain management-a multicenter prospective cohort study in pain clinics with one-year follow-up Pain Med (2019) 20 736–746. doi: 10.1093/pm/pny128, 30010966

[13] Liu CH Fu TS Lee CP Hung CI. Reliability and validity of the depression and somatic symptoms scale among patients with chronic low Back pain. Neuropsychiatr Dis Treat. (2019) 15:241–6. doi: 10.2147/ndt.S188277, 30679910 PMC6338111

[14] Hruschak V Cochran GJP, health, medicine. Psychosocial predictors in the transition from acute to chronic pain: a systematic review. (2018) 23:1151–1167. doi: 10.1080/13548506.2018.1446097 PMC6708443 29490476

[15] Chaichana KL Mukherjee D Adogwa O Cheng JS McGirt MJ. Correlation of preoperative depression and somatic perception scales with postoperative disability and quality of life after lumbar discectomy. J Neurosurg Spine. (2011) 14:261–7. doi: 10.3171/2010.10.Spine10190, 21214315

[16] Sinikallio S Aalto T Airaksinen O Herno A Kröger H Viinamäki H. Depressive burden in the preoperative and early recovery phase predicts poorer surgery outcome among lumbar spinal stenosis patients: a one-year prospective follow-up study. Spine. (2009) 34:2573–8. doi: 10.1097/BRS.0b013e3181b317bd, 19927107

[17] Wilhelm M Reiman M Goode A Richardson W Brown C Vaughn D . Psychological predictors of outcomes with lumbar spinal fusion: a systematic literature review. Physiother Res Int. (2017) 22. doi: 10.1002/pri.1648, 26270324

[18] Kim EJ Chotai S Stonko DP Wick JB Schneider BJ McGirt MJ . Patient-reported outcomes after lumbar epidural steroid injection for degenerative spine disease in depressed versus non-depressed patients. Spine J. (2017) 17:511–7. doi: 10.1016/j.spinee.2016.10.017, 27777051

[19] Lindemann C Hölzl A Böhle S Zippelius T Strube P. How does anxiety and depression affect the outcome after periradicular infiltration therapy?-a retrospective analysis of patients undergoing Ct-guided single-level nerve root infiltration due to chronic monoradicular pain. Diagnostics. (2023) 13. doi: 10.3390/diagnostics13182882, 37761249 PMC10527802

[20] Nagel B Gerbershagen H Lindena G MJDS P. Development and evaluation of the multidimensional German pain questionnaire. Schmerz. (2002) 16:263–70. doi: 10.1007/s00482-002-0162-1 12192435

[21] Casser H Hüppe M Kohlmann T Korb J Lindena G Maier C . Deutscher Schmerzfragebogen (Dsf) Und Standardisierte Dokumentation Mit Kedoq-Schmerz. Schmerz. (2012) 26:168–75. doi: 10.1007/s00482-011-1142-0, 22527646

[22] Margraf J Schneider S Ehlers A DiNardo P Barlow D. Dips Diagnostisches Interview Bei Psychischen Störungen: Interviewleitfaden. Berlin: Springer-Verlag (2013).

[23] Herrmann-Lingen C Buss U Snaith RJH. Hads-D Hospital Anxiety and Depression Scale-German Version. Mannheim: Huber Verlag (2011).

[24] Bjelland I Dahl AA Haug TT DJJopr N. The validity of the hospital anxiety and depression scale: an updated literature review (2002) 52:69–77. doi: 10.1016/S0022-3999(01)00296-3, 11832252

[25] Spinhoven P Ormel J Sloekers P Kempen G Speckens AE van Hemert AMJP. A validation study of the hospital anxiety and depression scale (Hads) in different groups of Dutch subjects (1997) 27:363–70. doi: 10.1017/S0033291796004382, 9089829

[26] Osman A Wong JL Bagge CL Freedenthal S Gutierrez PM Lozano GJJ. The depression anxiety stress scales—21 (Dass-21): further examination of dimensions, scale reliability, and correlates (2012) 68:1322–38. doi: 10.1002/jclp.21908, 22930477

[27] Coker AO Coker O Sanni DJARR. Psychometric properties of the 21-item depression anxiety stress scale (Dass-21). Afr Res Rev. (2018) 12:135–42. doi: 10.4314/afrrev.v12i2.13

[28] Nicholas MK Linton SJ Watson PJ Main CJ. Early identification and management of psychological risk factors ("yellow flags") in patients with low back pain: a reappraisal. Phys Ther. (2011) 91:737–53. doi: 10.2522/ptj.20100224 21451099

[29] Waddell G Main CJ Morris EW Di Paola M Gray IC. Chronic low-Back pain, psychologic distress, and illness behavior. Spine. (1984) 9:209–13. Epub 1984/03/01. doi: 10.1097/00007632-198403000-00013, 6233714

[30] Bener A Verjee M Dafeeah EE Falah O Al-Juhaishi T Schlogl J . Psychological factors: anxiety, depression, and somatization symptoms in low back pain patients. J Pain Res. (2013) 6:95–101. doi: 10.2147/jpr.S40740, 23403693 PMC3569050

[31] Manchikanti L Pampati V Beyer C Damron K Barnhill RC. Evaluation of psychological status in chronic low Back pain: comparison with general population. Pain Physician. (2002) 5:149–55. doi: 10.36076/ppj.2002/5/149, 16902665

[32] Thom J Kuhnert R Born S Hapke U. 12-Monats-Prävalenz Der Selbstberichteten Ärztlich Diagnostizierten Depression in Deutschland. Berlin: Robert Koch Institute (2017).

[33] Jacobi F Höfler M Siegert J Mack S Gerschler A Scholl L . Twelve-month prevalence, comorbidity and correlates of mental disorders in Germany: the mental health module of the German health interview and examination survey for adults (Degs1-Mh). Int J Methods Psychiatr Res. (2014) 23:304–19. doi: 10.1002/mpr.1439, 24729411 PMC6878234

[34] Arnold B Lutz J Nilges P Pfingsten M Rief W Böger A . Chronische Schmerzstörung Mit Somatischen Und Psychischen Faktoren (F45.41). Schmerz. (2017) 31:555–8. doi: 10.1007/s00482-017-0251-9, 29143118

[35] Nilges P Rief W. F45.41: chronic pain disorder with somatic and psychological factors: a coding aid. Schmerz. (2010) 24:209–12. doi: 10.1007/s00482-010-0908-0, 20372936

[36] Jindal R Rudol G Okafor B Rambani R. Role of psychological distress screening in predicting the outcomes of epidural steroid injection in chronic low Back pain. J Clin Orthop Trauma. (2021) 19:26–33. doi: 10.1016/j.jcot.2021.04.027, 34046297 PMC8141939

[37] Stensland M McGeary D Covell C Fitzgerald E Mojallal M Lugosi S . The role of psychosocial factors in mediating the treatment response of epidural steroid injections for low Back pain with or without lumbosacral radiculopathy: a scoping review. PLoS One. (2025) 20:e0316366. doi: 10.1371/journal.pone.0316366, 39813271 PMC11734955

[38] Karp JF Yu L Friedly J Amtmann D Pilkonis PA. Negative affect and sleep disturbance may be associated with response to epidural steroid injections for spine-related pain. Arch Phys Med Rehabil. (2014) 95:309–15. doi: 10.1016/j.apmr.2013.09.007, 24060493 PMC4008542

[39] Stensland M Sanford E Houle TT McGeary C Cobos BA Lugosi S . The relationship between psychosocial factors and response to epidural steroid injection for chronic lumbosacral radicular pain: a prospective pilot study. J Pain Res. (2025) 18:1991–2002. doi: 10.2147/jpr.s496290, 40241817 PMC12000912

